# Long-term results of regenerative treatment of intrabony defects: a cohort study with 5-year follow-up

**DOI:** 10.1186/s12903-023-03820-3

**Published:** 2024-01-06

**Authors:** Jae-Hong Lee, Seong-Nyum Jeong

**Affiliations:** 1 Department of Periodontology, College of Dentistry, Institute of Oral Bioscience; Research Institute of Clinical Medicine of Jeonbuk National University-Biomedical Research Institute of Jeonbuk National University Hospital, Jeonbuk National University, 567 Baekje-daero, Deokjin-gu, Jeonju, 54896 Korea; 2 Department of Periodontology, Daejeon Dental Hospital, Institute of Wonkwang Dental Research, Wonkwang University College of Dentistry, Daejeon, Korea

**Keywords:** Bone regeneration, Bone substitutes, Enamel matrix proteins, Periodontitis

## Abstract

**Background:**

The aim of this retrospective cohort study was to evaluate the long-term clinical and radiographic outcomes and survival of teeth in periodontal regenerative treatment of intrabony defects using combined enamel matrix protein derivative (EMD) and deproteinized porcine bone mineral (DPBM) compared to EMD alone.

**Methods:**

A total of 333 intrabony defects in 176 patients (mean age: 54.7 ± 8.9 years) were followed-up for 58.6 ± 11.2 (range, 25–78) months after periodontal regenerative treatment. Changes in clinical (pocket probing depth and clinical attachment level) and radiographic (defect depth and defect width) parameters were analyzed using serial periapical radiographs. Kaplan–Meier and multivariate Cox proportional-hazards regression analyses for tooth loss were also performed.

**Results:**

Compared to periodontal surgery with EMD alone with a mean follow-up of 5 years, combined EMD and DPBM showed significantly better gain in clinical attachment level (EMD and DPBM: 2.8 ± 2.3 mm vs. EMD alone: 2.2 ± 2.2 mm) and reduction in probing pocket depth (EMD and DPBM: 2.8 ± 1.8 mm vs. EMD alone: 2.3 ± 1.8 mm), defect depth (EMD and DPBM: 2.5 ± 2.4 mm vs. EMD alone: 2.0 ± 2.4 mm) and defect width (EMD and DPBM: 0.6 ± 1.0 mm vs. EMD alone: 0.2 ± 1.3 mm). The overall survival rates of the teeth were 91.48% and 95.20% in the patient- and tooth-based analyses, respectively, showing no statistically significant difference.

**Conclusions:**

Within the limitations of the current study, combined EMD and DPBM offered additional clinical and radiographic benefits over a mean of 5 years compared to EMD alone. However, tooth loss did not differ significantly between the two groups.

**Clinical relevance:**

Compared to EMD alone, combined EMD and DPBM for intrabony defects has additional clinical advantages; however, patient- and tooth-related risk factors must be considered when performing periodontal regenerative surgery.

## Introduction

Periodontal disease is a chronic infectious disease of the oral cavity with a prevalence of around 50%, which typically leads to destruction of the periodontal tissues and tooth loss [1–3]. Risk factors affecting the onset and deterioration of periodontal disease include modifiable factors, such as smoking, diabetes mellitus, oral pathologic microorganisms, and psychological stress, and non-modifiable factors, such as age, genetic factors, and host immune response [4, 5]. In addition, periodontal disease shares risk factors unidirectionally and bidirectionally with major chronic systemic diseases, including cardiovascular disease, diabetes mellitus, hypertension, rheumatoid arthritis, and osteoporosis [6–8]. Therefore, periodontitis-related complications are a growing public health concern associated with a high morbidity burden worldwide [9, 10].

Non-surgical and surgical periodontal procedures are widely used highly predictive treatment techniques, and their primary therapeutic goal is to maintain natural teeth and related soft and hard tissues functionally and healthily for a long time period [11]. In particular, clinical studies on various periodontal regenerative procedures, including guided tissue regeneration (GTR), bone grafts, and enamel matrix protein derivatives (EMDs), have shown a tooth survival rate of over 90% and reported that periodontal conditions are successfully treated and stably maintained for over 10 years [12, 13]. A recent long-term cohort study reported a tooth loss rate of 2.6% and improved mean defect fill that was sustained for 10 years after periodontal regenerative surgery of intra-bony defects [14]. Another long-term study confirmed that a tooth survival rate of 90% was achieved over a period of 13 years of functional loading and that clinical improvements were maintained at a rate of 82% for 11 years [15].

Despite the development of efficient treatment modalities and innovative materials, periodontal tissue regeneration remains challenging. [16] Although many clinical and epidemiological studies have confirmed that periodontal regenerative treatment shows better clinical and radiographic improvements compared to open flap debridement (OFD), long-term evidence of the benefits of periodontal regenerative treatment remain to be accumulated. [17, 18] Furthermore, there is limited long-term evidence to support the additional benefits of using deproteinized porcine bone mineral (DPBM) for periodontal defect regeneration. [19, 20] Therefore, the purpose of this cohort study was to evaluate the long-term clinical and radiographic outcomes and survival of teeth in periodontal regenerative treatment of intrabony defects using combined EMD and DPBM compared to EMD alone.

## Materials and methods

### Ethics

The study protocol was approved by the research ethics board at Daejeon Dental Hospital, Wonkwang University (approval No. W2208/003 − 001), and written informed consent was obtained from all patients before beginning of the study. The study was performed in accordance with the revised principles of the Helsinki Declaration and STROBE guidelines for the conduct and reporting of observational studies [21, 22]. All methods in this study were performed in accordance to relative guidelines and regulations.

### Patients

In this retrospective cohort study, patients who underwent periodontal regenerative surgery with EMD with or without adjunctive use of DPBM between September 2016 and December 2020 at the Department of Periodontology, Daejeon Dental Hospital, Wonkwang University were screened and reviewed. The EMD alone group and the combined EMD and DPBM group were determined based on the additional cost of using bone graft substitutes and the patient’s personal choice. Inclusion criteria were: (1) age ≥ 19 years; (2) presence of intrabony defects treated with regenerative surgery; (3) 0 or 1 degree of tooth mobility before regenerative surgery; 4)stable periodontal status (full-mouth bleeding-on-probing and plaque scores < 25%); 5) systemically healthy or controlled medical condition; and 6) follow-up after periodontal surgery ≥ 2 years. Exclusion criteria were: (1) heavy smoking (≥ 20 cigarettes/day); (2) uncontrolled systemic diseases or periodontal conditions; (3) intrabony defects extending into the furcation region (grade II or III); and (4) no or irregular supportive periodontal treatment (SPT).

**Fig. 1:**
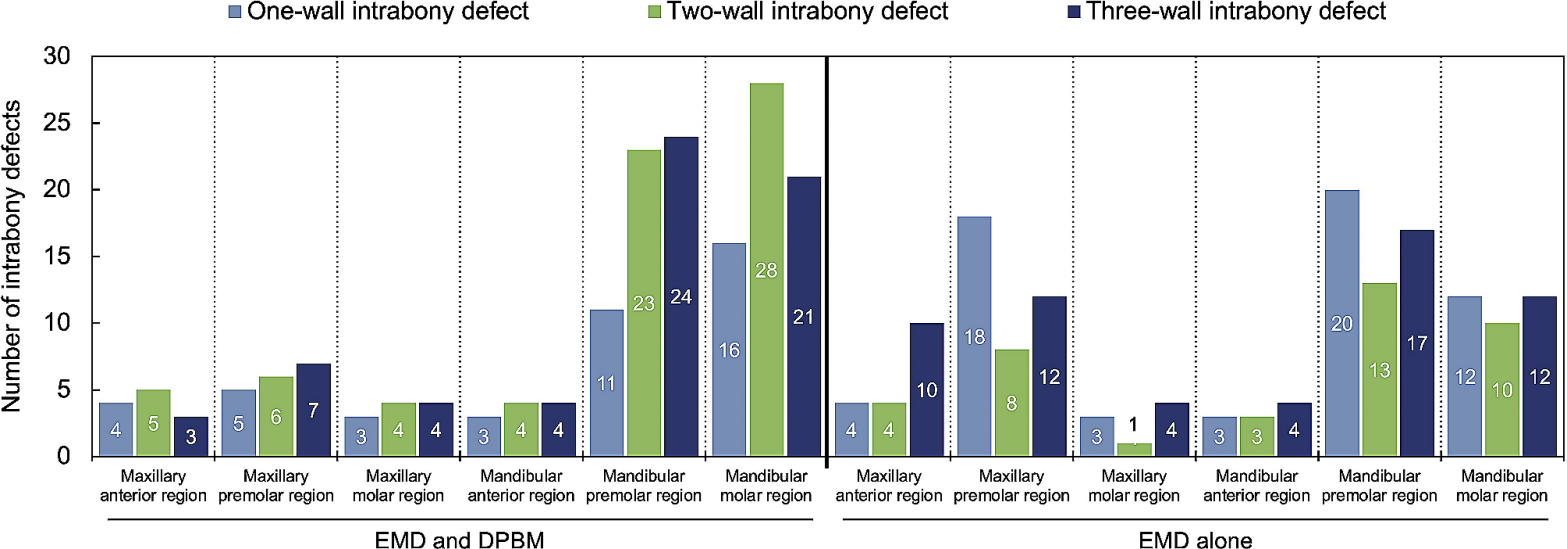
Frequency distribution of intrabony defects according to the tooth position

The present cohort included 176 patients with 333 intrabony defects (mean 1.9 defects/patient), comprising 115 (65.3%) males and 61 (34.7%) females, with a mean age of 54.7 ± 8.9 (range, 25–80) years at T0. We found seven (4.0%) cases of diabetes mellitus, 30 (17.0%) cases of hypertension, 156 (88.6%) non-smokers, and 20 (11.4%) smokers with < 20 cigarettes/day. The mean follow-up duration was 58.6 ± 11.2 (range, 25–78) months. The distribution of defect morphology according to the number of walls showed a statistically significant difference between the compared two groups (*p* = 0.003). Table 1 shows the detailed baseline information. The intrabony defects were distributed as follows: maxillary anterior region, *n* = 30 (9.0%); maxillary premolar region, *n* = 56 (16.8%); maxillary molar region, *n* = 19 (5.7%); mandibular anterior region, *n* = 21 (6.3%); mandibular premolar region, *n* = 108 (32.4%); and mandibular molar region, *n* = 99 (29.7%) (Fig. 1.).

**Table 1:** Baseline characteristics of involved patients and treatment sites

	EMD and DPBM (mean 1.3 defects/patient)			EMD alone (mean 3.7 defects/patient)		*p*-value
	*n*	%		*n*	%	
Number of patients	133	100.0		43	100.0	
Sex						
Male	84	63.2		31	72.1	0.285
Female	49	36.8		12	27.9	
Age (mean ± SD, years)	55.4 ± 8.9			52.8 ± 8.9		0.106
Diabetes mellitus	3	2.3		4	9.3	0.040^*^
Hypertension	24	18.0		6	14.0	0.536
Smoking status						
Non-smoker	115	86.5		41	95.3	0.111
Current smoker (< 20 cigarettes per day)	18	13.5		2	4.7	
Follow-up time (mean ± SD, months)	58.5 ± 11.2			58.9 ± 11.2		0.863
Number of treatment sites	175	100.0		158	100.0	
Defect morphology						
One-wall	42	24.0		60	38.0	0.003^**^
Two-wall	70	40.0		39	24.7	
Thress-wall	63	36.0		59	37.3	

EMD, enamel matrix protein derivative; DPBM, deproteinized porcine bone mineral; SD, standard deviation

Variables of significance (^*^p ≤ 0.05 and ^**^p ≤ 0.01)

### Surgical regenerative procedure

A board-certified periodontal specialist (J.H.L.) performed all surgeries. Under local anesthesia (2% lidocaine, 1:100,000 epinephrine), a full-thickness mucoperiosteal flap was minimally elevated to access the intrabony defect using simplified or modified papilla preservation techniques [23, 24]. Granulation tissues were removed, and the exposed tooth surfaces were scaled and planed with an ultrasonic scaler (SONICflex air scaler, KaVo, Biberach, Germany) and manual curettes (standard and mini Gracey curettes, Hu-Friedy, Chicago, USA). The debrided root surfaces were then conditioned with tetracycline hydrochloride at a concentration of 50 mg/mL for 2 min and rinsed with a sterile saline solution. Subsequently, adequate amount of EMD (Straumann Emdogain® 0.3 mL, Straumann, Basel, Switzerland) was applied to the hemostatic and dried tooth surface and defect site, with or without the adjunctive use of DPBM (deproteinized porcine bone mineral, THE Graft® 0.25 g, Purgo Biologics, Seongnam, Korea). In the combined EMD and DPBM group, the remaining EMD and DPBM were mixed and then the defect site was evenly filled with a condenser and any excess EMD and DPBM was removed. Tension-free flap closure was performed with interrupted (absorbable 6–0 Vicryl®, Johnson & Johnson, New Jersey, and non-absorbable 3–0 Biotex®, Purgo, Seongnam, Korea) and horizontal mattress (non-absorbable 4–0 Dafilon®, Braun Surgical, Tuttlingen, Germany, and non-absorbable 3–0 Biotex®) sutures.”

### Post-surgical procedure

All treated patients received post-operative antibiotics (Amoxicillin®, Chongkundang Pharm, Seoul, Korea, amoxicillin 500 mg thrice daily) and analgesics (Brufen®, Samil Co, Seoul, Korea, ibuprofen 200 mg thrice daily) for 3–7 days and were instructed to rinse their mouths twice daily with 15 mL of 0.12% chlorhexidine digluconate (Hexamedine®, Bukwang Pharm, Seoul, Korea) for 1 min for 2 weeks. After 2 weeks since periodontal regeneration surgery, the sutures were removed, and the surgical site was cleansed with a sterile saline solution. For SPT, professional tooth cleaning, with provision of plaque control instructions, was performed every 3–6 months depending on the periodontal inflammatory status of each patient.

### Clinical and radiographic parameters

**Fig. 2:**
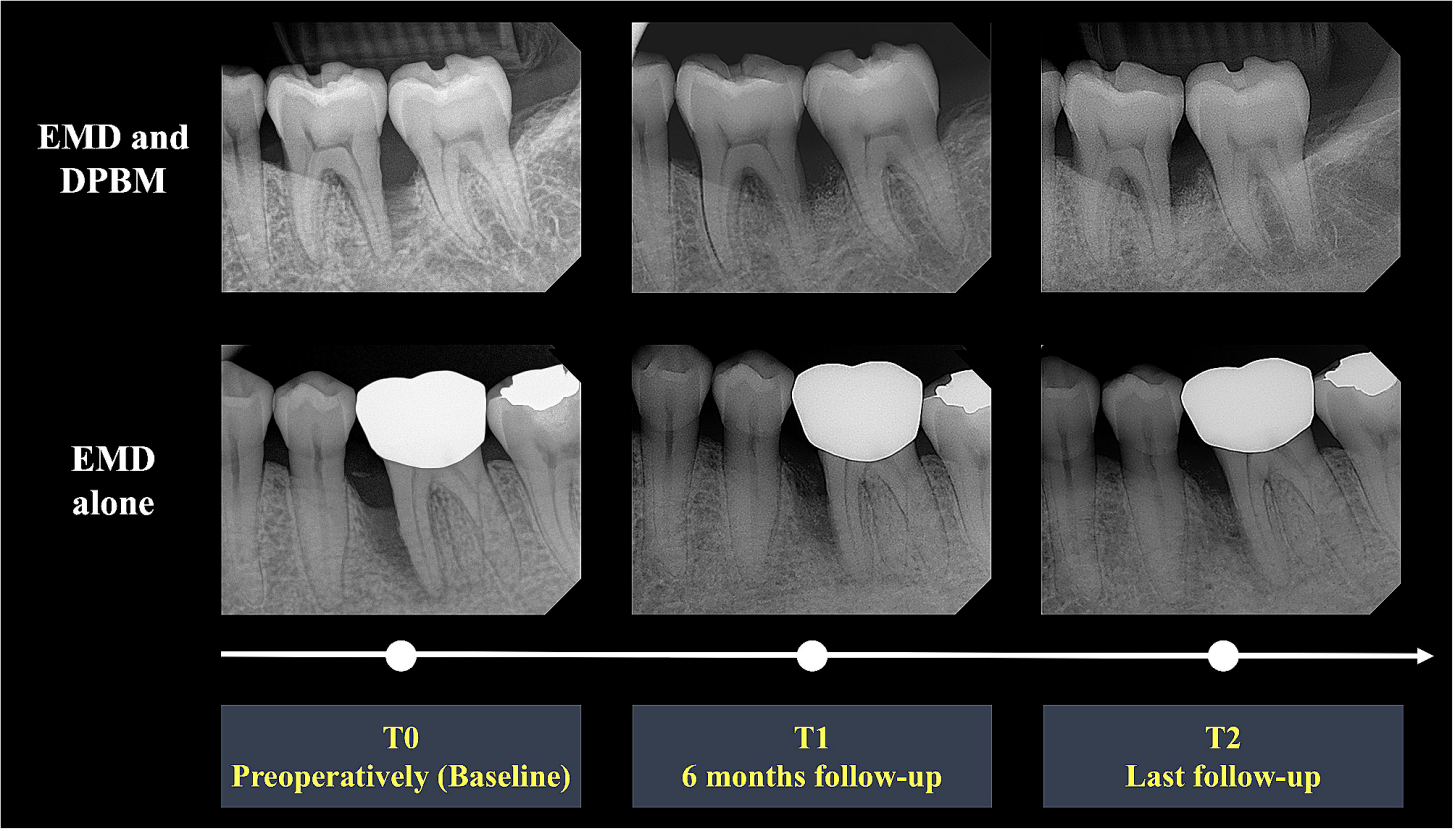
Schematic representation of periapical radiographs and time points

Clinical and radiographic parameters were measured at the baseline (T0, preoperatively), 6-month follow-up (T1), and last follow-up (T2) after regenerative surgery for intrabony defects. Clinical parameters included the probing pocket depth (PPD), measured as the vertical distance between the gingival margin and the bottom of the periodontal pocket, and clinical attachment level (CAL), measured as the vertical distance between the cementoenamel junction and the bottom of the periodontal pocket. Radiographic parameters included defect depth (DD), measured as the vertical distance from the alveolar crest to the bottom of the bone defect, and defect width (DW), measured as the horizontal distance from the alveolar crest to the root surface. A single calibrated examiner who was not involved in the surgery recorded clinical and radiographic parameters using a periodontal probe (CP 15 UNC, Hu-Friedy, Chicago, IL, USA) and medical imaging software (Osirix X version 12.5.3, Pixmeo SARL, Geneva, Switzerland) (Fig. 2.).

### Statistical analysis

Descriptive statistics are expressed as frequencies, proportions, mean, and standard deviation. An intra-examiner agreement test was performed to determine the reliability of the radiographic assessments. 10 cases were measured twice, and the intra-examiner correlation showed over 90% reproducibility by a single examiner who was not involved in the surgical procedures. The Shapiro–Wilk test was performed to assess normality of data distribution, and Levene’s test was used to assess the homogeneity of variances. Independent *t*-tests and paired *t*-tests were performed to identify significant differences in the clinical and radiographic parameters between and within the groups at T0, T1, and T2, respectively. Kaplan–Meier estimates were used to analyze the time to events for tooth loss over the observational period, and log-rank tests were conducted to compare survival curves of teeth treated with and without the adjunctive use of DPBM. The multivariate Cox proportional-hazards regression analysis adjusted for age, sex, smoking status, hypertension, diabetes mellitus, tooth position, defect morphology, and presence/absence of DPBM was used to assess the hazard ratio (HR) of the risk of tooth loss after periodontal regenerative surgery. All statistical analyses were conducted using statistical software (SPSS Statistics version 28.0, IBM Corp., Armonk, New York, and MedCalc version 20.114, Mariakerke, Belgium), and a *p*-value < 0.05 was considered to indicate statistical significance.

## Results

### Clinical and radiographic outcomes

In the combined EMD and DPBM group, the mean PPD and CAL changed significantly from 7.9 ± 1.9 mm at T0 to 5.2 ± 1.6 mm at T2 (mean difference [MD]: -2.8 ± 1.8 mm, *p* < 0.001) and 8.5 ± 2.1 mm at T0 to 5.8 ± 2.1 mm at T2 (MD: -2.8 ± 2.3 mm, *p* < 0.001), respectively. In the EMD alone group, the mean PPD and CAL changed significantly from 7.6 ± 1.5 mm at T0 to 5.3 ± 1.5 mm at T2 (MD: -2.3 ± 1.8 mm, *p* < 0.001) and 8.1 ± 1.9 mm at T0 to 5.9 ± 2.1 mm at T2 (MD: -2.2 ± 2.2 mm, *p* < 0.001), respectively. In the combined EMD and DPBM group, the mean DD and DW reduced significantly from 6.8 ± 2.6 mm at T0 to 4.3 ± 2.1 mm at T2 (MD: -2.5 ± 2.4 mm, *p* < 0.001) and 1.7 ± 1.0 mm at T0 to 1.1 ± 0.9 mm at T2 (MD: -0.6 ± 1.0 mm, *p* < 0.001), respectively. In the EMD alone group, the mean DD and DW reduced significantly from 6.6 ± 2.4 mm at T0 to 4.6 ± 1.9 mm at T2 (MD: -2.0 ± 2.4 mm, *p* < 0.001) and 1.7 ± 1.2 mm at T0 to 1.5 ± 1.2 mm at T2 (MD: -0.2 ± 1.3 mm, *p* = 0.093), respectively.

**Fig. 3:**
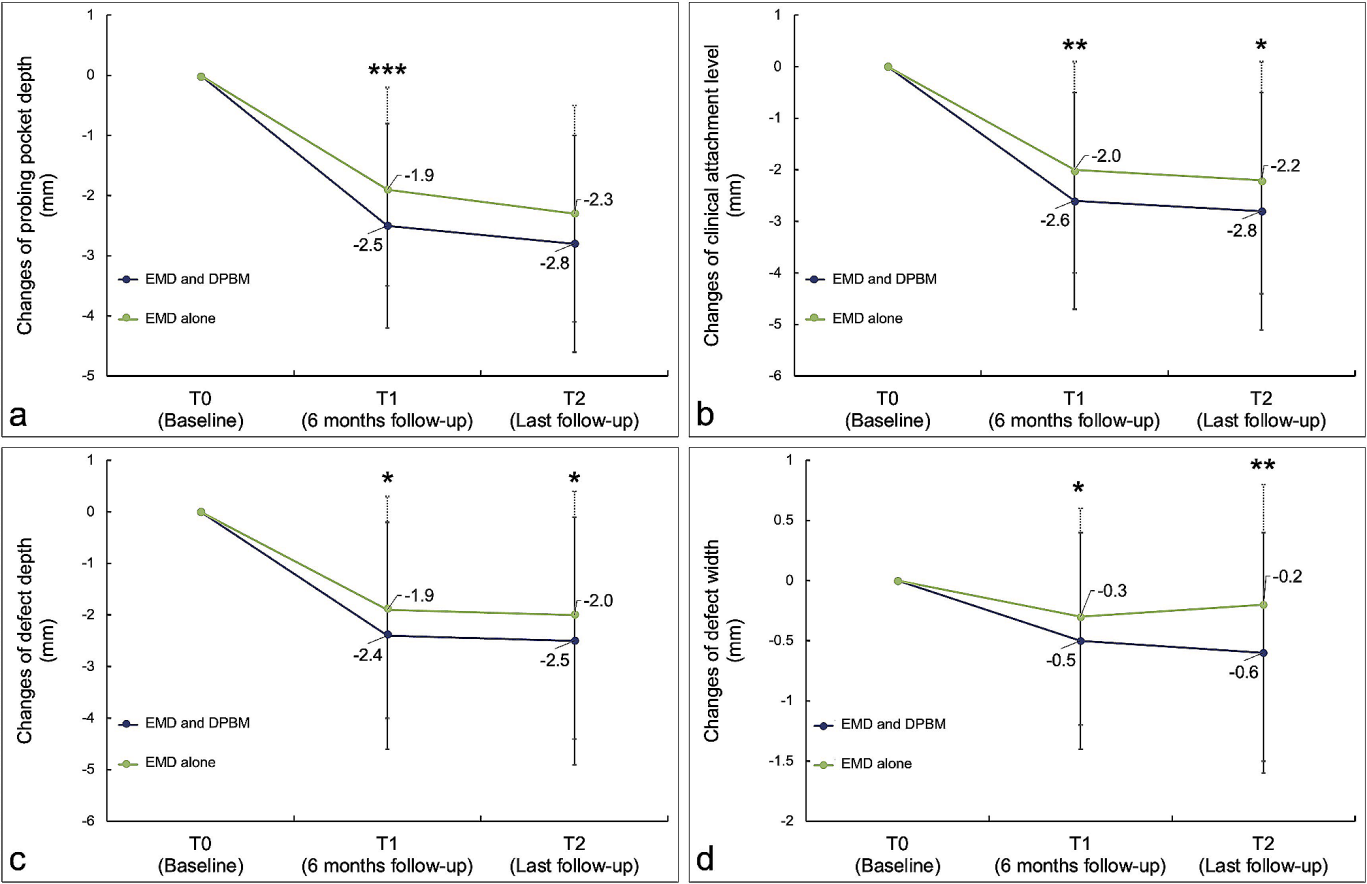
Changes in clinical (**a** and **b**) and radiographic (**c** and **d**) outcomes from the 6-months follow-up (T1) to the last follow-up (T2). Variables of significance (^*^p ≤ 0.05, ^**^p ≤ 0.01, and ^***^p ≤ 0.001)

Compared to periodontal surgery with EMD alone with a mean follow-up of 5 years, combined EMD and DPBM showed significantly better gain in CAL (EMD and DPBM: 2.8 ± 2.3 mm vs. EMD alone: 2.2 ± 2.2 mm, *p* = 0.019) and reduction in PPD (EMD and DPBM: 2.8 ± 1.8 mm vs. EMD alone: 2.3 ± 1.8 mm, *p* = 0.028), DD (EMD and DPBM: 2.5 ± 2.4 mm vs. EMD alone: 2.0 ± 2.4 mm, *p* = 0.040) and DW (EMD and DPBM: 0.6 ± 1.0 mm vs. EMD alone: 0.2 ± 1.3 mm, *p* = 0.007). Table 2; Fig. 3. provide detailed clinical and radiographic outcomes at T0, T1, and T2.

**Table 2:** Clinical and radiographic outcomes at the baseline (T0), 6-month follow-up (T1), and last follow-up (T2) after regenerative treatment of intrabony defects

Parameters (mm)	EMD and DPBM				EMD alone				*p*-value (EMD and DPBM vs. EMD alone)		
	T0	T1	T2		T0	T1	T2		T0–T1	T1–T2	T0–T2
Clinical outcomes											
PPD											
Total	7.9 ± 1.9 (7.6–8.2)	5.4 ± 1.7^***^ (5.2–5.7)	5.2 ± 1.6 (4.9–5.4)		7.6 ± 1.5 (7.4–7.9)	5.8 ± 1.5^***^ (5.5–6.0)	5.3 ± 1.5^**^ (5.1–5.5)		0.001	0.115	0.028
One-wall	9.0 ± 2.0 (8.4–9.6)	6.7 ± 1.3^***^ (6.3–7.1)	6.0 ± 1.6 (5.8–6.9)		8.3 ± 1.7 (7.8–8.7)	6.8 ± 0.9^***^ (6.6–7.0)	6.1 ± 1.3^**^ (5.7–6.4)		0.031	0.718	0.078
Two-wall	7.7 ± 1.8 (7.3–8.1)	5.4 ± 1.5^***^ (5.0–5.7)	5.1 ± 1.4 (5.5–6.9)		7.4 ± 1.5 (6.9–8.0)	5.6 ± 1.4^***^ (5.1–6.1)	5.2 ± 1.4 (4.7–5.7)		0.036	0.308	0.239
Three-wall	7.4 ± 1.7 (7.0–7.8)	4.6 ± 1.6^***^ (4.3–5.0)	4.6 ± 1.5 (5.0–6.5)		7.1 ± 1.1 (6.8–7.4)	4.8 ± 1.5^***^ (4.4–5.2)	4.6 ± 1.4 (4.3–5.0)		0.112	0.414	0.354
CAL											
Total	8.5 ± 2.1 (8.2–8.8)	6.0 ± 2.1^***^ (5.7–6.3)	5.8 ± 2.1 (5.5–6.1)		8.1 ± 1.9 (7.8–8.4)	6.1 ± 2.0^***^ (5.8–6.4)	5.9 ± 2.1 (5.6–6.2)		0.008	0.864	0.019
One-wall	9.5 ± 2.1 (8.9–10.1)	7.9 ± 1.6^***^ (7.4–8.4)	7.6 ± 1.8 (7.1–8.1)		8.8 ± 1.8 (8.3–9.2)	7.5 ± 1.3^***^ (7.1–7.8)	6.9 ± 1.7 (6.5–7.4)		0.580	0.367	0.880
Two-wall	8.4 ± 2.0 (7.9–8.9)	5.9 ± 1.7^***^ (5.5–6.3)	5.6 ± 1.7 (6.0–7.7)		8.0 ± 1.9 (7.4–8.7)	5.7 ± 1.8^***^ (5.1–6.3)	5.5 ± 1.8 (4.8–6.2)		0.744	0.518	0.517
Three-wall	8.1 ± 1.9 (7.6–8.5)	4.7 ± 1.9^***^ (4.2–5.2)	4.8 ± 2.0 (5.3–7.3)		7.4 ± 1.7 (6.9–7.8)	5.0 ± 2.0^***^ (4.5–5.5)	5.1 ± 2.1 (4.5–5.6)		0.001	0.854	0.003
Radiographic outcomes											
Defect depth											
Total	6.8 ± 2.6 (6.5–7.2)	4.5 ± 2.1^***^ (4.2–4.8)	4.3 ± 2.1 (4.0–4.6)		6.6 ± 2.4 (6.2–7.0)	4.7 ± 1.9^***^ (4.4–5.0)	4.6 ± 1.9 (4.3–4.9)		0.034	0.799	0.040
One-wall	7.6 ± 3.2 (6.7–8.6)	4.8 ± 2.6^***^ (4.0–5.6)	4.4 ± 2.7 (3.6–5.2)		7.7 ± 2.8 (7.0–8.4)	5.8 ± 1.8^***^ (5.3–6.2)	5.6 ± 1.7 (5.2–6.0)		0.065	0.557	0.037
Two-wall	6.7 ± 2.4 (6.2–7.3)	5.0 ± 1.7^***^ (4.6–5.4)	4.9 ± 1.6 (5.3–6.9)		5.8 ± 2.2 (5.0–6.5)	4.7 ± 1.7^**^ (4.0–5.3)	4.6 ± 1.7 (4.0–5.2)		0.160	0.830	0.148
Three-wall	6.4 ± 2.2 (5.9–6.9)	3.6 ± 1.8^***^ (3.1–4.0)	3.5 ± 1.8 (4.0–5.8)		6.1 ± 1.6 (5.7–6.5)	3.7 ± 1.7^***^ (3.3–4.1)	3.6 ± 1.8 (3.1–4.1)		0.124	0.867	0.296
Defect width											
Total	1.7 ± 1.0 (1.5–1.8)	1.2 ± 1.0^***^ (1.0–1.3)	1.1 ± 0.9 (1.0–1.2)		1.7 ± 1.2 (1.6–1.9)	1.4 ± 1.1 (1.3–1.6)	1.5 ± 1.2 (1.3–1.7)		0.067	0.102	0.007
One-wall	1.9 ± 1.0 (1.6–2.2)	1.5 ± 1.2 (1.1–1.9)	1.4 ± 1.0 (1.1–1.7)		1.9 ± 1.2 (1.7–2.2)	1.8 ± 0.8 (1.6–2.0)	2.0 ± 1.2 (1.7–2.3)		0.179	0.119	0.028
Two-wall	1.6 ± 0.9 (1.4–1.8)	1.3 ± 0.9^**^ (1.1–1.5)	1.1 ± 0.7 (1.3–1.9)		1.5 ± 0.6 (1.3–1.7)	1.2 ± 0.3^**^ (1.1–1.3)	1.0 ± 0.3^**^ (0.9–1.1)		0.835	0.731	0.957
Three-wall	1.6 ± 1.1 (1.3–1.8)	0.8 ± 0.9^***^ (0.6–1.1)	0.9 ± 1.1 (1.1–2.2)		1.7 ± 1.4 (1.3–2.0)	1.2 ± 1.4 (0.8–1.6)	1.3 ± 1.4 (1.0–1.7)		0.170	0.653	0.136

CAL: clinical attachment level; PPD: probing pocket depth

Data are expressed as mean ± standard deviation (95% confidence interval)

*p*-values for comparisons between (1) T0 and T1 and (2) T1 and T2 and variables of significance (^*^p ≤ 0.05, ^**^p ≤ 0.01, and ^***^p ≤ 0.001)

### Tooth survival outcomes

**Fig. 4:**
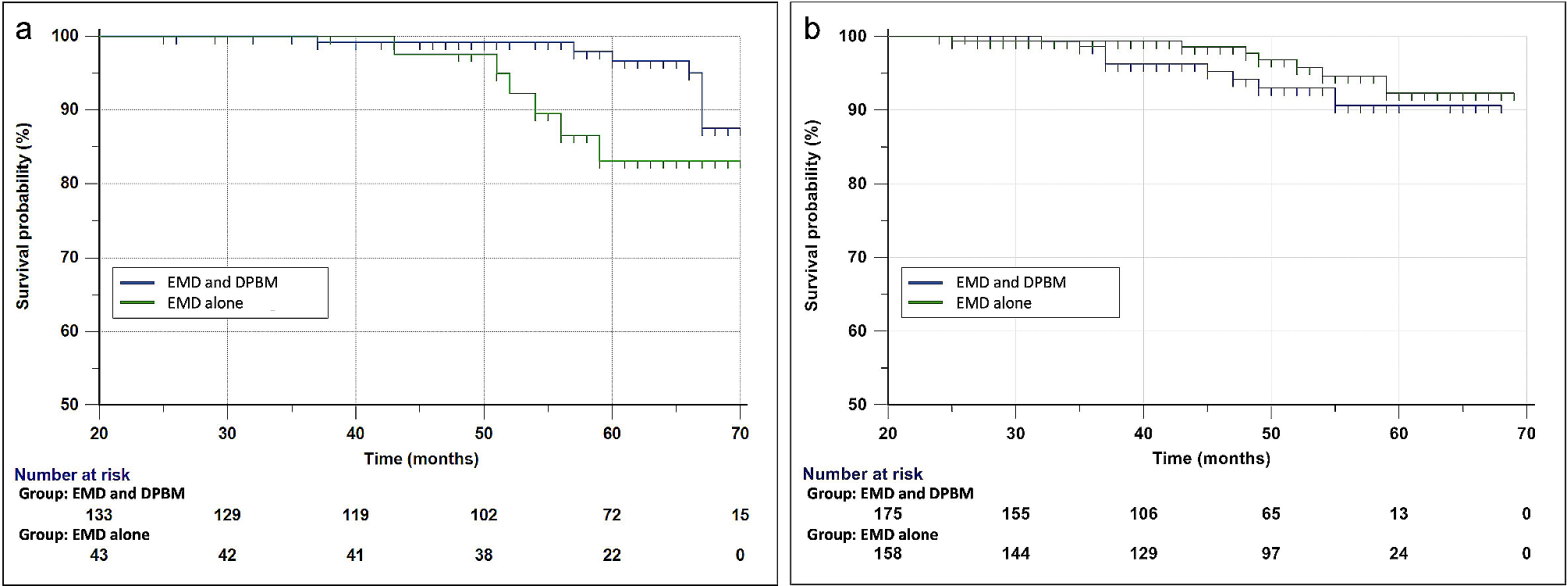
Tooth survival analysis. Kaplan–Meier survival estimates grouped by porcine-derived xenograft use in (**a**) patient- and (**b**) tooth-based analyses show survival rates of 91.48% and 95.20% over the follow-up period, respectively

A total of 16 teeth of nine (56.3%) male and seven (43.8%) female, with a mean age of 56.3 ± 7.8 (range, 42–68) years, were lost due to severe mobility, recurrence of pain, and signs of infection during the follow-up period. Most (*n* = 10, 62.5%) teeth were lost in the one-wall intrabony defect, followed by two-wall (*n* = 4, 25.0%) and three-wall (*n* = 2, 12.5%) intrabony defects. The mean follow-up time until tooth loss was 58.2 ± 10.0 (range, 37–74) months (Table 3). The overall survival rate of teeth did not differ between the compared two groups. At the end of the study period, the survival rates of the teeth were 91.48% and 95.20% in the patient- and tooth-based analyses, respectively. Figure 4. shows the Kaplan–Meier estimates of tooth survival. The multivariate Cox proportional-hazards regression analysis for tooth loss after adjusting for age, sex, smoking status, hypertension, diabetes mellitus, tooth position, defect morphology, and DPBM use showed that tooth loss after periodontal regenerative treatment had a significant positive association with diabetes mellitus (reference: no diabetes mellitus, HR = 44.57, *p* = 0.003), the maxillary molar region (reference: maxillary anterior region, HR = 13.08, *p* = 0.022), and one-wall intrabony defects (reference: three-wall intrabony defect, HR = 18.73, *p* = 0.002; Table 4).

**Table 3:** Characteristics of tooth loss

Patient characteristics						Surgery				Tooth loss	
Case number	Age (years)	Sex	Smoking status	Systemic disease		Tooth number	With xenograft	Defect morphology		Reason for tooth loss	Duration before tooth loss (months)
1	52	Female	No	N-S		36	Yes	One-wall		Recurrence of pain and signs of infection	37
2	57	Male	Yes	N-S		41	No	One-wall		Severe mobility	43
3	59	Male	No	HTN		16	Yes	Two-wall		Recurrence of pain and signs of infection	49
4	60	Male	No	N-S		17	No	One-wall		Recurrence of pain and signs of infection	51
5	44	Female	No	N-S		47	No	Two-wall		Recurrence of pain and signs of infection	52
6	50	Female	No	DM		37	No	One-wall		Recurrence of pain and signs of infection	54
7	42	Female	No	DM, HTN		11	No	Three-wall		Severe mobility	56
8	45	Male	No	N-S		36	Yes	Two-wall		Root fracture	57
9	61	Female	No	N-S		46	No	Two-wall		Recurrence of pain and signs of infection	59
10	68	Female	No	N-S		33	Yes	Three-wall		Recurrence of pain and signs of infection Severe mobility	60
11	63	Male	No	N-S		11	Yes	One-wall		Recurrence of pain and signs of infection	66
12	62	Male	No	HTN		37	Yes	One-wall		Recurrence of pain and signs of infection Severe mobility	67
13	67	Male	No	HTN		46	Yes	One-wall		Recurrence of pain and signs of infection Severe mobility	67
14	51	Male	No	N-S		26	Yes	One-wall		Recurrence of pain and signs of infection Severe mobility	67
15	61	Female	No	N-S		27	Yes	One-wall		Recurrence of pain and signs of infection	72
16	59	Male	No	HTN		16	No	One-wall		Severe mobility	74

DM, diabetes mellitus; HTN, hypertension; N-S, non-specific (absence of systemic diseases that specifically affect periodontal disease, including hypertension and diabetes)

**Table 4:** Multivariate Cox proportional hazards regression analyses for risk factors potentially affecting the tooth loss

variable	hazard ratio	std. error	95% Confidence interval		z-score	*P*-value
Age (reference: 20–29 years)						
30–39	0.00	0.00	0.00	-	0.00	0.999
40–49	0.08	856.95	0.00	-	0.00	1.000
50–59	0.03	367.45	0.00	-	0.00	1.000
60–69	0.18	1,915.84	0.00	-	0.00	1.000
70–79	0.00	0.00	0.00	-	0.00	0.999
80–89	0.74	14,722.86	0.00	-	0.00	1.000
Sex (reference: male)						
Female	1.32	1.03	0.29	6.11	0.36	0.720
Smoking status (reference: non-smoker)						
Current smoker	3.08	3.19	0.40	23.51	1.08	0.278
Hypertension (reference: no)						
Yes	4.17	3.23	0.91	19.06	1.84	0.065
Diabetes mellitus (reference: no)						
Yes	44.57	57.48	3.56	558.20	2.94	0.003^**^
Position (reference: maxillary anterior)						
Maxillary premolar	0.00	0.00	0.00	-	-0.01	0.994
Maxillary molar	13.08	14.64	1.46	117.24	2.30	0.022^*^
Mandibular anterior	2.55	3.07	0.24	27.12	0.77	0.438
Mandibular premolar	0.00	0.00	0.00	-	-0.01	0.992
Mandibular molar	1.38	1.35	0.20	9.36	0.33	0.741
Defect morphology (reference: three-wall)						
One-wall	18.73	17.43	3.02	116.09	3.15	0.002^**^
Two-wall	4.93	4.96	0.69	35.41	1.58	0.113
With xenograft (reference: no)						
Yes	0.75	0.48	0.21	2.63	-0.45	0.653

Variables of significance (^*^p ≤ 0.05 and ^**^p ≤ 0.01)

The multivariate Cox proportional-hazards regression analysis adjusted for age, sex, smoking status, hypertension, diabetes mellitus, tooth position, defect morphology, and with or without porcine-derived xenograft

## Discussion

The objective of this cohort study was to evaluate the long-term clinical and radiographic outcomes of periodontal regenerative treatment for intrabony defects using EMD with and without DPBM. Combined EMD and DPBM showed significantly better clinical and radiographic outcomes, consistent with previous studies demonstrating that combined EMD and bone grafting improves the regeneration of periodontal intrabony and furcation defects [25, 26]. However, although most clinical studies has reported that regenerative therapy is a highly promising treatment strategy for periodontal defects compared to OFD, no clear consensus on the superiority or inferiority relationship among different regenerative treatment modalities has been reached [27, 28].

Periodontal regeneration surgery with EMD has additional benefits compared to OFD in treating intraosseous defects [29–31]. In a recent cohort study, periodontal regenerative surgery with EMD showed significantly changed the mean PPD and CAL from 6.71 ± 1.22 to 3.75 ± 1.41 mm (*p* < 0.001) and 8.43 ± 1.86 to 5.81 ± 1.83 mm (*p* < 0.001), respectively, and achieved a tooth survival rate of 90.7% over a mean observation period of 10.3 years [30]. In another longitudinal meta-analysis, the relative clinical value of periodontal regeneration therapies, including EMD and GTR, compared to OFD sustained up to 5–10 years [12]. Moreover, during the long follow-up period, clinical parameters, including PPD and CAL, did not differ statistically between the EMD and GTR groups [12].

In a previous systematic review and meta-analysis, combined EMD and bone grafts provided additional clinical benefits in terms of PD reduction (EMD and bone grafts: 4.22 ± 1.20 mm vs. EMD alone: 4.12 ± 1.07 mm) and CAL gain (EMD and bone grafts: 3.76 ± 1.07 mm vs. EMD alone: 3.32 ± 1.04 mm) compared to EMD alone. [13] However, in another recent meta-analytic review, combined EMD and bone grafts showed no statistically significant improvement in terms of PD reduction (standard difference in means [SDM]: -0.43 mm, *p* = 0.06) or CAL gain (SDM: -0.34 mm, *p* = 0.12) compared to EMD alone [32].

Various patient- and tooth-related factors can significantly influence tooth loss following active periodontal treatment [33, 34]. The oral hygiene status during SPT (risk ratio [RR] = 1.58, *p* < 0.001), irregular SPT (RR = 3.17, *p* < 0.001), initial diagnosis of periodontitis (RR = 2.33, *p* < 0.001), age (RR = 1.05, *p* < 0.001), smoking (RR = 1.80, *p* < 0.05), and sex (RR = 1.45, *p* < 0.05) were patient-related risk factors, and the baseline bone loss (odds ratio [OR] = 1.05, *p* < 0.001), furcation involvement (OR = 1.80, *p* < 0.05), and abutment tooth (OR = 1.80, *p* < 0.05) were tooth-related risk factors significantly contributing to tooth loss (tooth based survival rate: 93.26%) in Poisson and logistic multilevel regression analyses over 10 years [33, 34].

Within the limited research findings, compared to periodontal surgery with EMD alone, with a mean follow-up of 5 years, combined EMD and DPBM showed statistically significant better gains in CAL and reductions in PPD, DD and DW. However, the overall clinical outcomes showed that not only PPD but also CAL improved, but the results still showed mean PPD and CAL values greater than 5 mm. These results may be due to the fact that the analysis of this study included not only three-wall defects, but also non-contained one-wall and/or furcation defects and maxillary molar regions with a poor prognosis.

In the present study, the 5-year overall survival rates of the teeth were 91.48% and 95.20% in the patient- and tooth-based analyses, respectively, showing no statistically significant difference between the two compared groups in terms of the patient- or tooth-based survival rate. We also found that the loss of teeth showed a statistically significant association with diabetes mellitus (HR = 44.57, *p* = 0.003), the maxillary molar position (HR = 13.08, *p* = 0.022), and one-wall intrabony defects (HR = 18.73, *p* = 0.002), after adjusting for patient- and tooth-related confounding variables.

In addition to local risk factors, such as the tooth position, root divergence, and abutment tooth for fixed or partial dental prostheses, directly related to tooth loss, diabetes mellitus is a major risk factor for periodontal disease and tooth loss, and biologically plausible underlying mechanisms have been proposed [19, 20, 35–37]. Moreover, recent systematic reviews have reported a consistently high risk for complications of diabetes mellitus, such as diabetic retinopathy (OR = 2.8–8.7), neuropathy (OR = 3.2–6.6), nephropathy (OR = 1.9–8.5), cardiovascular complications (OR = 1.28–17.7), and mortality (OR = 2.3–8.5), in the presence of periodontal disease [38]. Therefore, in determining the outcomes of periodontal regenerative treatment, local tooth-related factors and the presence and control of diabetes mellitus must be considered.

In addition to intrabony defect morphology, the degree of furcation involvement is a major risk factor influencing the long-term outcomes and tooth mortality [39, 40]. According to a meta-analysis, the relative risk of tooth loss due to the presence of furcation involvement is 2.21 (95% confidence interval = 1.79–2.74, *p* < 0.001) up to 15 years of follow-up [39]. In a recent long-term retrospective cohort study, 37% of teeth with class III furcation involvement were lost over an average of 9 years after active periodontal treatment [40]. Therefore, the treatment sites included in this study were selected with the aim to minimize the effect of tooth loss due to furcation involvement by limiting intrabony defects associated with furcation involvement grade I.

Although significant clinical and radiographic improvements were observed with combined EMD and DPBM compared to EMD alone, the results of the present study should be interpreted with caution. First, although efforts have been undertaken to standardize treatment approaches, current study has an inherent limitation of a retrospective observational study design. Second, due to heterogeneity within or between groups, selective and informative biases should be considered when interpreting the findings. Third, as no calibrated or standardized methods were used for the radiographic measurements, caution should be taken when interpreting the reproducibility of the measurements. Fourth, previous studies have reported clinical benefits of the adjunctive use of EMD in periodontal surgery in reducing post-operative pain, swelling, and soft tissue wound healing, but the relevant parameters were not measured in this study. Furthermore, the lack of a negative control group receiving only OFD is a major limitation. Therefore, further well-designed and bias-controlled clinical trials are required to apply our current findings in clinical practice and draw reliable conclusions.

## Conclusion

Within the aforementioned limitations, the results of this study indicated that combined EMD and DPBM may result in significant additional clinical and radiographic improvements in terms of PPD, CAL, DD, and DW compared to EMD alone over a mean follow-up of 5 years. However, tooth loss did not differ significantly between the two compared groups. Further well-controlled prospective trials of long-term outcomes are necessary to confirm our findings.

## Data Availability

The datasets collected and analyzed during the current study are not publicly available due to legal/ethical reasons but are available from the corresponding author on reasonable request.
